# Network Analysis and Precision Rehabilitation for the Post-concussion Syndrome

**DOI:** 10.3389/fneur.2019.00489

**Published:** 2019-05-29

**Authors:** Grant L. Iverson

**Affiliations:** ^1^Department of Physical Medicine and Rehabilitation, Harvard Medical School, Boston, MA, United States; ^2^Spaulding Research Institute, Spaulding Rehabilitation Hospital, Charlestown, MA, United States; ^3^MassGeneral Hospital for Children Sport Concussion Program, Boston, MA, United States; ^4^Home Base, A Red Sox Foundation and Massachusetts General Hospital Program, Charlestown, MA, United States

**Keywords:** concussion, traumatic brain injury, rehabilitation, post-concussional syndrome, depression

## Abstract

Some people experience persistent symptoms following a mild traumatic brain injury (MTBI), and the etiology of those symptoms has been debated for generations. Post-concussion-like symptoms are caused by many factors both before and after MTBI, and this non-specificity is the bedrock of the conundrum regarding the existence of the post-concussion syndrome. A latent model or common cause theory for the syndrome is inconsistent with the prevailing biopsychosocial conceptualization. It is the thesis of this paper that adopting a network perspective for persistent symptoms following MTBI, including the post-concussion syndrome, could lead to new insights and targeted treatment and rehabilitation strategies. The network perspective posits that symptoms co-occur because they are strongly inter-related, activating, amplifying, and mutually reinforcing, not because they arise from a common latent disease entity. This approach requires a conceptual shift away from thinking that symptoms reflect an underlying disease or disorder toward viewing inter-related symptoms as constituting the syndrome or disorder. The symptoms do not arise from an underlying syndrome—the symptoms are the syndrome. A network analysis approach allows us to embrace heterogeneity and comorbidity, and it might lead to the identification of new approaches to sequenced care. The promise of precision rehabilitation requires us to better understand the interconnections among symptoms and problems so that we can produce more individualized and effective treatment and rehabilitation.

## Introduction

A substantial minority of people report persistent symptoms following a mild traumatic brain injury (MTBI) for several months and sometimes years (1–9). Whether these symptoms represent a “post-concussion syndrome” has been controversial for generations. For decades, researchers, and clinicians have questioned whether this diagnosis is a true syndrome, disorder, or disease entity [e.g., (10–15)], and the etiology of the syndrome has never been agreed upon [see (16–21) for reviews]. Many have suggested that the etiology of persistent symptoms is due to the biological effects of a MTBI, psychological factors, psychosocial factors (broadly defined), chronic pain, depression, or a combination of factors (22–30). Regardless of etiology, persistent symptoms after MTBI are associated with high levels of disability and health care service utilization, and lower health-related quality of life (9, 31–40).

The International Classification of Diseases 10th edition (ICD-10) specific research criteria for the post-concussional syndrome require symptoms to be present for more than 1 month and the person must have symptoms and problems in three or more of the following domains (1) complaints of unpleasant sensations and pains, such as headache, dizziness (usually lacking the features of true vertigo), general malaise and excessive fatigue, or noise intolerance; (2) emotional changes, such as irritability, emotional lability, both easily provoked or exacerbated by emotional excitement or stress, or some degree of depression and/or anxiety; (3) subjective complaints of difficulty in concentration and in performing mental tasks, and of memory complaints, without clear objective evidence (e.g., psychological tests) of marked impairment; (4) insomnia; (5) reduced tolerance to alcohol; and/or (6) preoccupation with the above symptoms and fear of permanent brain damage, to the extent of hypochondriacal over-valued ideas and adoption of a sick role (41). The Diagnostic and Statistical Manual of Mental Disorders-Fourth Edition (DSM-IV) (42) included research criteria for the post-concussional disorder that differed from the ICD-10 criteria in a several ways, such as including somewhat different symptoms and requiring the presence of objectively measured cognitive deficits. For the 5th Edition (i.e., the DSM-5), published in 2013 (43), the post-concussional disorder was dropped and problems relating to MTBI can be coded as “mild neurocognitive disorder,” but this diagnosis does not include post-concussion symptoms—it is based on objective evidence of a decline in cognitive functioning.

A fundamental challenge in defining the syndrome is the non-specificity of the symptoms. Post-concussion-like symptoms are common in healthy children and adults in their daily lives (44–51). They are also common in people seen for psychological treatment (52), outpatients seen for minor medical problems (53), personal injury claimants (53, 54), and people with post-traumatic stress disorder (PTSD) (55), orthopedic injuries (11), chronic pain (42, 56–59), whiplash (60), anxiety (61, 62), and depression (63). Biopsychosocial conceptualizations of the symptoms and syndrome (64–66) emphasize a diverse range of personality and social psychological factors that contribute to how symptoms are perceived, experienced, and reported, such as expectations and misattributions (47, 67–71), coping and illness perceptions (72), “good-old-days” bias (47, 73–79), cognitive hypochondriasis (80), fear avoidance (81, 82) cogniphobia (83, 84), nocebo effect (85, 86), perceived injustice (87), iatrogenesis (17, 27), resilience (88, 89), Type D personality (90, 91), and other personality characteristics, particularly compulsive, histrionic, dependent, and narcissistic traits (21, 61). A multidimensional model for conceptualizing the post-concussion syndrome suggests that setbacks in several aspects of a person's life (physical, emotional, cognitive, psychosocial, vocational, financial, and recreational) serve as cumulative stressors that interact with personality and pre-morbid physical and mental health factors, resulting in the syndrome (21, 92, 93). Clearly, post-concussion-like symptoms are caused by many factors both before and after a mild injury to the brain. This non-specificity problem incumbers the development of new and innovative approaches to conceptualizing the etiology of symptoms and developing new treatment and rehabilitation strategies.

**Figure 1:**
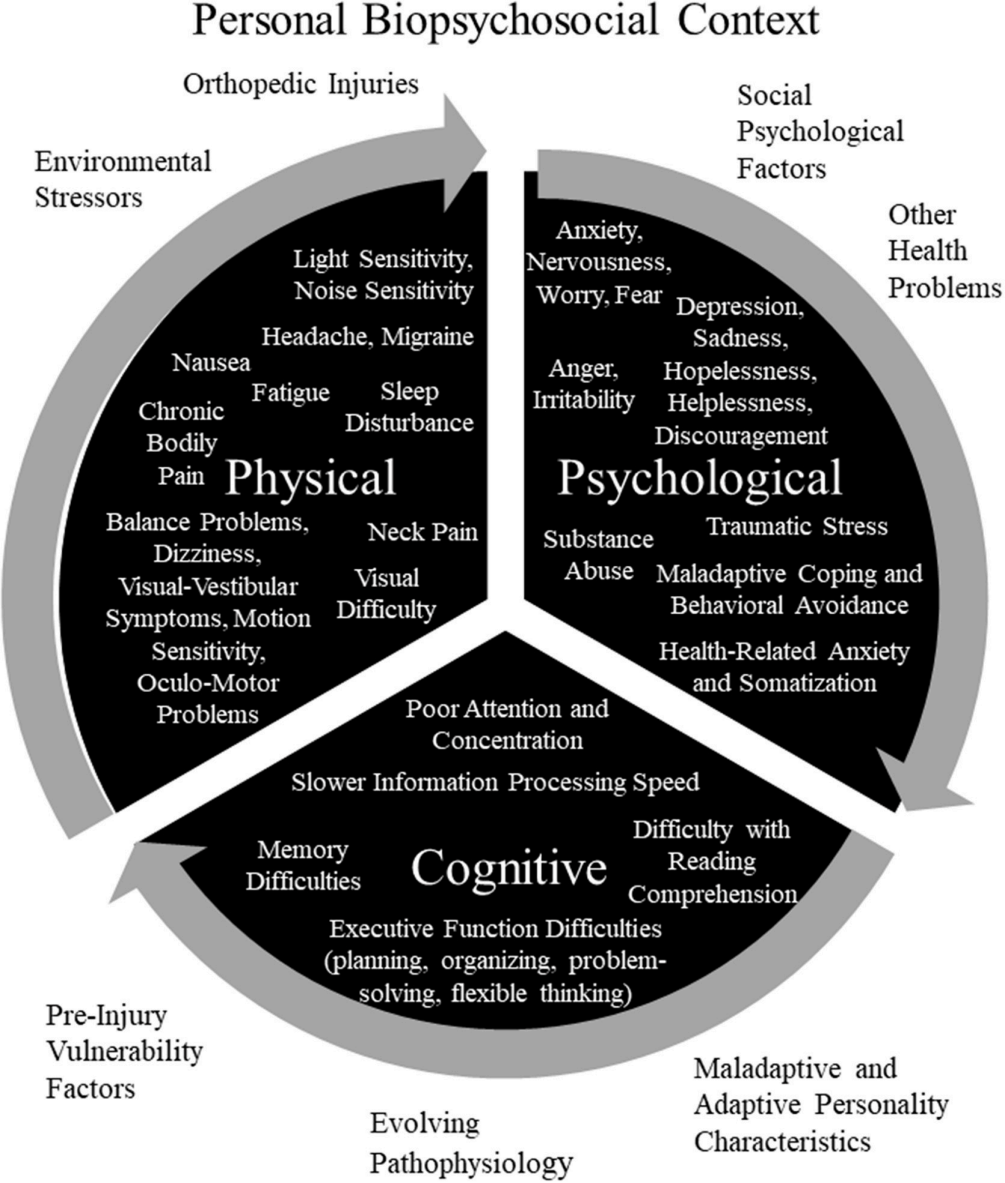
Potentially Amplifying and Reinforcing Persistent Symptoms and Problems and Personal Biopsychosocial Context for Experiencing Persistent Symptoms and Problems. Pre-Injury Vulnerability Factors: personal or family history of mental health problems and associated genetic and environmental vulnerability (childhood abuse or neglect, depression, anxiety, or traumatic stress); prior brain injuries; personal history of, or vulnerability to, migraine or other headache disorder; and history of motion sickness or other visual-vestibular vulnerability factor. Environmental Stressors: financial/occupational stress; academic stress; marital, family, or relationship problems; and litigation, compensation-seeking or maintaining, or other secondary gain issues. Social Psychological Factors: maladaptive coping, catastrophizing, expectations, “good-old-days” bias (tendency to view oneself as healthier in the past and underestimate past problems), nocebo effect, diagnosis threat, cognitive hypochondriasis and preoccupation, lifestyle and family dynamics changes, avoidance behavior, cogniphobia (fear and avoidance of mental exertion out of concern for developing or exacerbating a headache), reinforced illness behavior, anger, bitterness, perceived injustice, justification/entitlement, or iatrogenesis. Personality Characteristics or Disorders: neuroticism (a personality trait characterized by a strong tendency to experience negative emotions such as anxiety, depression, anger, and self-consciousness. Individuals with this trait have considerable difficulty coping with stress), anxiety sensitivity (a trait comprised of physical, psychological, and social pre-occupations and concerns, is characterized by fear of anxiety-related bodily sensations), alexithymia (a cluster of traits characterized by difficulty identifying feelings, difficulty describing feelings to others, externally oriented thinking, and limited capacity for imaginal thinking), perfectionism, egocentrism, Type D personality (personality pattern is characterized by two stable personality traits: negative affectivity and social inhibition), disagreeableness (a personality trait characterized by antagonism, skepticism, and egocentrism), unconscientiousness (a trait characterized by reduced self-discipline and ambition, disorganization, and a more lackadaisical approach to life), narcissistic, dependent, histrionic, or passive-aggressive. Adaptive Personality Characteristics: resilience, grit (passion and perseverance toward long-term goals), and psychological hardiness (personality characteristic consisting of three psychological attitudes and beliefs: commitment, challenge, and control). Copyright © 2019, Grant L. Iverson, Ph.D., Used with Permission.

As seen in Figure 1, a diverse array of physical, psychological, and cognitive symptoms and problems can be amplifying and mutually reinforcing in people who have experienced a mild TBI. Some of those symptoms might be caused directly or indirectly by injuries to the brain, head, peripheral vestibular system, or body, and some symptoms might be caused, amplified, or maintained by a diverse range of other factors. It is essential to appreciate that these diverse symptoms and problems occur within a *personal biopsychosocial context*, as seen in Figure 1. Individuals have unique pre-injury vulnerability factors, current environmental stressors, social psychological reactionary factors, and personality characteristics. As such, an individual's symptoms and problems (from Figure 1) occur within a unique personal context. The symptoms that a person experiences, their underlying causes, and the biopsychosocial context in which the person lives all are subject to change over time, from the initial days following injury to weeks and months later.

The biopsychosocial heterogeneity and complexity associated with outcome from MTBI is illustrated further in an interesting review by Kenzie et al. (94). They propose a conceptual framework, involving multiple nested scales, based on a complex systems theoretical approach. The four nested scales are cellular (e.g., axonal injury, neuroinflammation, and synaptic changes), network (e.g., intrinsic connectivity and neuronal population dynamics), experiential (e.g., physical, cognitive, and psychological symptoms), and social (e.g., access to healthcare, social support, work or school pressures)—and each of these interacting scales can be influenced by a diverse range of personal characteristics and external environmental factors [see Figure 1 in Kenzie et al. (94)]. Given the complexity of persistent symptoms following a mild injury to the brain, as reflected in the conceptual model of Kenzie and colleagues and in Figure 1, it is not surprising that there is no unified latent disease model etiology for the post-concussion syndrome.

## Network Analysis and Persistent Post-Concussion Symptoms

It is the thesis of this paper that adopting a network perspective for persistent symptoms following MTBI, including the post-concussion syndrome, could lead to new insights and targeted treatment and rehabilitation strategies. To my knowledge, there are no published studies applying network analysis to persistent symptoms and problems following MTBI. Network theory and analysis (95–100) posits that mental disorders can be viewed as a set of interacting symptoms. Conceptually and philosophically, the network approach does not require that the post-concussion syndrome, or syndromes, have a single underlying cause (e.g., brain injury) that is *independent* of the symptoms. Instead, the presence of the interacting and inter-related symptoms *constitutes* the syndrome. A syndrome may occur when a requisite number of symptoms become activated for a sufficient period of time. The network approach, applied to the post-concussion syndrome, posits that symptoms co-occur because they are strongly inter-related, activating, amplifying, and mutually reinforcing, not because they arise from a common latent disease entity. This approach requires a conceptual shift away from thinking that symptoms *reflect* an underlying disease or disorder toward viewing inter-related symptoms as *constituting* the syndrome or disorder. That is, the symptoms are the syndrome.

## Network Analysis in Psychiatry and Psychology

Network analysis is a statistical and psychometric methodology for studying the interrelationships among symptoms. A number of articles describe the methodology of network analysis (96, 101, 102). A network, graphically represented, is comprised of nodes and edges. For the purpose of this paper, a node is a symptom or clinical feature of PCS, and the edges are connections between symptoms. The edges represent the statistical associations between symptoms. When represented as a figure, symptoms (i.e., nodes) that activate each other are connected by lines (i.e., edges). The interrelations among the symptoms represent the network. Specific symptoms within the network can be influenced by things in the “external field.” The external field is outside the symptom network, but not necessarily outside the person. The external field is comprised of intrinsic (e.g., microstructural brain injury, cervical injury, or peripheral vestibular injury) and extrinsic factors (e.g., life stress). The external field also includes the social and environmental context for the person, including being involved in personal injury litigation or a worker's compensation claim.

**Figure 2:**
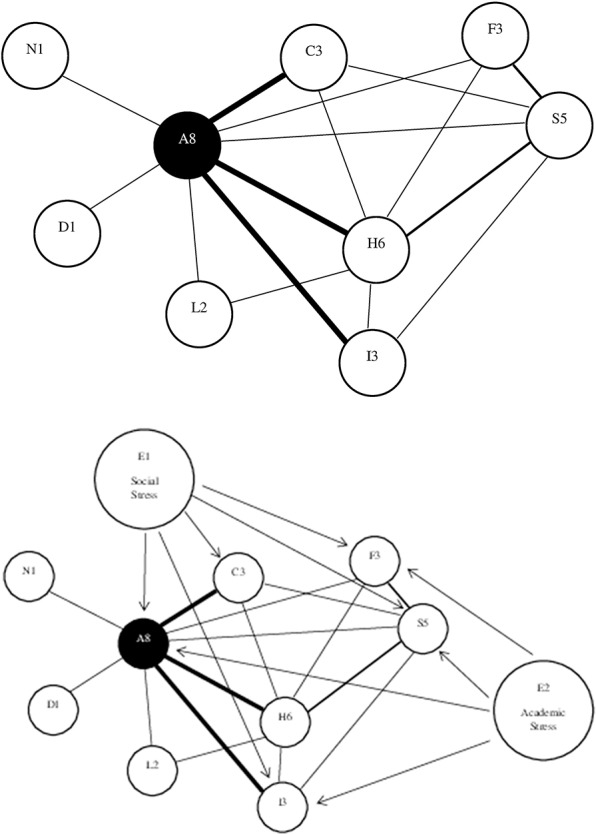
*Hypothetical* network of nine symptoms in slow to recover high school and college students with pre-existing anxiety problems. The top figure shows that anxiety (A8) has the greatest degree (eight connections to other symptoms) and strength (three heavier lines) of centrality, followed by headaches (H6) and sleep (S5). Three symptoms are connected to three other symptoms (nodes): irritability (I3), concentration problems (C3), and fatigue (F3). Light sensitivity (L2) is connected to two other symptoms, and nausea (N1) and dizziness (D1) are connected to only one other symptom. The bottom figure illustrates the role of external factors, in the external field, that are amplifying the network of symptoms, such as social stress (E1) and academic stress (E2).

A network of symptoms is represented graphically, in a two-dimensional figure, by having circles representing symptoms and lines connecting circles representing the association (i.e., correlation) between the symptoms. These graphic depictions arise from statistical psychometric analyses of large databases, not from theory. The lines can be unweighted, which means that all statistically significant correlations are shown with the same thickness of lines, or they can be weighted, meaning that thicker lines represent stronger correlations. Line colors can also be used, such as green lines representing positive associations and red lines representing negative associations. The lines connecting the symptoms can be undirected (no arrows) or directed (one arrow). A directed line (i.e., edge) shows the hypothetical direction of the association between symptoms (e.g., symptom A activates symptom B). See Figure 2 for a *hypothetical* network of nine symptoms in slow to recover high school and college students with pre-existing anxiety problems. Symptom *centrality* is an important concept in the network analysis. Central symptoms are those that are most important in the network, and there are several was to measure centrality including the degree, strength, expected influence, closeness, and betweenness. It is important to appreciate that a limitation of network analysis diagrams is that they can lead to the impression that inter-related and interacting between symptoms are static, when in fact they might be temporally sequenced and dynamic.

Fried and colleagues reviewed the literature on network analysis in psychology and psychiatry (95). Network analysis has been used to better understand the structure of emotional and behavioral problems in children (103), the central symptoms and syndromic pathways of traumatic stress in children and adolescents (104–106), longitudinal developmental associations between symptoms of depression and anxiety (107), and the associations between internalizing and externalizing psychopathology in the transition from childhood to adolescence (107). In adults, syndromic pathways between social anxiety, perceived stress, and problematic alcohol use have been identified (108). In fact, network analysis has been used to simultaneously study 12 major psychiatric diagnoses in a sample of more than 34,000 adults, with the resulting network illustrating differential associations between symptoms within the same diagnosis and strong connections with symptoms from other diagnoses, illuminating the complexity of psychopathology and psychiatric comorbidity (100). Because depression and PTSD are so common in civilians, military service members, and veterans who have sustained MTBIs and who report long-term symptoms and problems, some recent advances in those areas, based on a network empirical and theoretical perspective, are discussed in the sections below.

### Depression

Pre-injury mental health problems, such as depression and anxiety, are a risk factor for persistent symptoms following MTBI (21, 36, 109–112). Depression is common following TBIs of all severities (113–115). Depression is also common in people with chronic pain (116–119), chronic headaches (120–123), PTSD (55, 124–126), and substance abuse problems (127–131). Primary depression can mimic the post-concussion syndrome (132), and depression has a very large effect on post-concussion-like symptom reporting (48, 63, 132–134). Moreover, post-injury worry, stress, and anxiety are thought to be central features of long-term symptom reporting (17, 27, 41, 61).

Network analysis is leading to important new insights in depression (135–139). Depression can be viewed as a complex dynamic system of interacting symptoms, some of which are core syndromal symptoms of depression (e.g., sadness and anhedonia) and some of which are not (e.g., anxiety and sympathetic arousal) (137). It is well established in psychiatry that depression and anxiety are comorbid in many people (140), and cross-sectional network analysis studies have illustrated *how* major depressive disorder and generalized anxiety disorder are interconnected, entangled, and amplifying (141–143). Moreover, chronic pain and depression often co-occur (116–119), and new studies are examining associations between symptom networks in chronic pain and depression (144), and how self-efficacy, fear avoidance, and perceived disability might link the pain experience with affective disorder symptoms (145).

Network analysis has been used to better understand the course of illness and the probability of relapse. People in remission from a prior episode of depression are at increased risk for developing future depression, and resilience is central and important for successfully coping with stressors and maintaining good mental health (146). Moreover, transitional states from being healthy to being depressed are not well-understood. “Critical slowing” (147) is a phenomenon in depression characterized by dynamic networks of symptoms taking increasingly longer to adapt or recover to perturbations, eventually leading to a tipping point into the development of a syndrome. The concept of critical slowing is applicable to a pathway by which a person might develop persistent symptoms following an MTBI.

### Post-traumatic Stress Disorder

Traumatic stress is fairly common in both civilians and military personnel who sustain MTBIs (148, 149). People with PTSD report symptoms that overlap with the post-concussion syndrome, such as irritability, cognitive problems, and sleep disturbance (55), and PTSD might have an amplifying or additive effect on symptom reporting following MTBI (150, 151). Network analysis has been used in diverse studies of PTSD (152–157). Researchers have used network analysis to examine (i) how specific combinations of symptoms might drive the development of PTSD in trauma-exposed adults (154); (ii) whether traumatic stress symptom presentations vary in association with different types of index traumas (158); (iii) the symptom connectivity and associations in combat veterans with PTSD and subthreshold PTSD (159, 160), and the interactions among traumatic stress symptoms, suicidal ideation, depression, and quality of life in veterans (153); (iv) the association between PTSD and alcohol use disorders (161); (v) the identification of central symptoms and bridging symptoms relating to the comorbidity of PTSD and major depressive disorder (162), and (vi) the comorbidity of GAD, depression, and PTSD (163). It is believed that better understanding of which symptoms of traumatic stress are more central and strongly interconnected than others may have implications for targeting clinical interventions.

## Conclusions and Directions for Future Research

It has long been believed by some researchers that no central underlying disease mechanism for the post-concussion syndrome has ever been found because it does not exist. The longstanding challenge for conceptualizing the post-concussion syndrome is that the constellation of symptoms comprising the syndrome are non-specific. Therefore, it is difficult to accept that post-concussion symptoms cohere as a syndrome because they share a single latent underlying cause, such as brain damage or a mental disorder. Multiple social psychological factors, such as expectations and misattributions (47, 67–71), “good-old-days” bias (47, 73–79), perceived injustice (87), fear avoidance (81, 82), socio-environmental factors such as compensation seeking (164–166), vulnerability factors such as pre-injury mental health problems (21, 36, 109–111), and neurological factors, such as microstructural changes to white matter (167), have been shown to be associated with persistent post-concussion symptoms. However, none have emerged as a latent common cause, and most in the field accept that post-concussion symptoms are multifactorial in causation. A latent model or common cause theory for the syndrome is inconsistent with a biopsychosocial conceptualization (64–66).

A network perspective makes it possible to study the architecture of persistent symptoms and problems following MTBI, allowing the identification of symptoms that are more central and strongly interconnected. A network perspective allows us to embrace the challenges of heterogeneity, non-specificity, comorbidity, and the latent disease model that have plagued the field of mild neurotrauma. The network perspective eschews the idea that a single latent dimension is the underlying cause of both symptom emergence and coherence. Symptoms can be causally connected through diverse biopsychosocial mechanisms. Network theory is agnostic with regard to how causal relations among symptoms are exemplified. The direct causal associations between some symptoms might be predominately biological, whereas other symptoms might have associations that are more strongly psychological. Symptoms can form amplification and self-sustaining feedback loops. If the inter-relations among the symptoms are strong enough, the symptoms become entrenched, self-sustaining, and in combination they comprise and represent a syndrome. In this context, a post-concussion syndrome does not exist separately from the symptoms that constitute it. In fact, a network perspective might identify *multiple syndromes*, or at least clusters of prominent symptoms, that might occur in subgroups of people following MTBI.

Adopting a network perspective in clinical research might help us identify single, paired, or small clusters of strongly interconnected symptoms that could be initial targets for treatment and rehabilitation (168). In the hypothetical example set out in Figure 2, an aggressive treatment and rehabilitation strategy targeting the two most central symptoms, anxiety, and headaches, might dampen the amplifying inter-relations among multiple symptoms leading to improvement across the entire network of symptoms. In theory, and of particular relevance to sequenced care following MTBI, targeting one or two symptoms with a high degree of strength of centrality might dampen or even ameliorate other symptoms in the acute or subacute period following injury, potentially preventing entrenchment and persistence of symptoms. It might also help us better understand complex comorbidities, such as depression, anxiety, PTSD, chronic pain, peripheral vestibular problems, and substance abuse, how they are inter-related, and how they might bridge and amplify post-concussion-like symptoms. Future research using network analysis might reveal syndrome profiles and inter-relations with comorbidities that could be targets for time-sequenced precision rehabilitation—leading to more personalized health care. Precision rehabilitation requires us to better understand the interconnections among symptoms and problems so that we can produce more effective treatment and rehabilitation for them.

## Author Contributions

The author confirms being the sole contributor of this work and has approved it for publication.

### Conflict of Interest Statement

The author has been reimbursed by the government, professional scientific bodies, and commercial organizations for discussing or presenting research relating to MTBI and sport-related concussion at meetings, scientific conferences, and symposiums. He has a clinical practice in forensic neuropsychology involving individuals who have sustained mild TBIs (including athletes). He has received honorariums for serving on research panels that provide scientific peer review of programs. He is a co-investigator, collaborator, or consultant on grants relating to mild TBI funded by the federal government and other organizations. He has received research support from test publishing companies in the past, including ImPACT^®^ Applications Systems, Psychological Assessment Resources, and CNS Vital Signs. He has received grant funding from the National Football League and salary support from the Harvard Integrated Program to Protect and Improve the Health of NFLPA Members. He serves as a scientific advisor for BioDirection, Inc., SWAY Operations, LLC, and Highmark, Inc.
